# Consequences, conditions and caveats: a qualitative exploration of the influence of undergraduate health professions students at distributed clinical training sites

**DOI:** 10.1186/s12909-018-1412-y

**Published:** 2018-12-19

**Authors:** Susan van Schalkwyk, Julia Blitz, Ian Couper, Marietjie de Villiers, Guin Lourens, Jana Muller, Ben van Heerden

**Affiliations:** 1 Centre for Health Professions Education, Faculty of Medicine and Health Sciences, Stellenbosch University, Stellenbosch, South Africa; 2 Ukwanda Centre for Rural Health, Faculty of Medicine and Health Sciences, Stellenbosch University, Stellenbosch, South Africa; 3 Family Medicine and Primary Care, Faculty of Medicine and Health Sciences, Stellenbosch University, Stellenbosch, South Africa; 4 MB,ChB Unit, Faculty of Medicine and Health Sciences, Stellenbosch University, Stellenbosch, South Africa

**Keywords:** Distributed clinical training, Community based education, Qualitative study, Undergraduate health professions training

## Abstract

**Background:**

Traditionally, the clinical training of health professionals has been located in central academic hospitals. This is changing. As academic institutions explore ways to produce a health workforce that meets the needs of both the health system and the communities it serves, the placement of students in these communities is becoming increasingly common. While there is a growing literature on the student experience at such distributed sites, we know less about how the presence of students influences the site itself. We therefore set out to elicit insights from key role-players at a number of distributed health service-based training sites about the contribution that students make and the influence their presence has on that site.

**Methods:**

This interpretivist study analysed qualitative data generated during twenty-four semi-structured interviews with facility managers, clinical supervisors and other clinicians working at eight distributed sites. A sampling grid was used to select sites that proportionally represented location, level of care and mix of health professions students. Transcribed data were subjected to thematic analysis. Following an iterative process, initial analyses and code lists were discussed and compared between team members after which the data were coded systematically across the entire data set.

**Results:**

The clustering and categorising of codes led to the generation of three over-arching themes: influence on the facility (culturally and materially); on patient care and community (contribution to service; improved patient outcomes); and on supervisors (enriched work experience, attitude towards teaching role). A subsequent stratified analysis of emergent events identified some consequences of taking clinical training to distributed sites. These consequences occurred when certain conditions were present. Further critical reflection pointed to a set of caveats that modulated the nature of these conditions, emphasising the complexity inherent in this context.

**Conclusions:**

The move towards training health professions students at distributed sites potentially offers many affordances for the facilities where the training takes places, for those responsible for student supervision, and for the patients and communities that these facilities serve. In establishing and maintaining relationships with the facilities, academic institutions will need to be mindful of the conditions and caveats that can influence these affordances.

## Background

A major driver for the Flexnerian reforms in medical education over a century ago was the need to align training more closely with service delivery. Recent calls from the Lancet Commission on educating health professionals for the twenty-first Century [1] are even more vocal about the importance of that link. Traditionally, health professions training has taken place in central academic hospitals. Distributed approaches that enable the training of health professions students away from these central facilities are becoming increasingly common, as academic institutions consider ways to produce a health workforce that meets the needs of the system and ultimately of the community it serves [2, 3]. This shift has necessitated a review of the nature of the required collaborative relationship between academic institutions and health services. There is growing evidence of health services embracing the training of students as crucial to their future survival [4]. In some cases, the valuable role played by students has been recognised in established health service-medical school partnerships [5–7]. However, questions as to the benefit of having students at distributed sites are still raised by health service managers, with some feeling that students present a drain on resources and/or negatively influence service delivery and patient care [3].

Much of what we know about the impact of students on the health facility when training occurs outside of tertiary care is anecdotal. There is a need for more evidence on which to base future health service-university partnerships. Specifically, data with regard to experiences resulting from the placement of a range of health care professions students and across all levels of care would be of value. The potential roles of students are numerous, ranging from contributions to direct patient care at the point of presentation, to longitudinal community outreach and quality improvement initiative activities [5]. It has been argued that we may never be able to quantify the value of such roles, but that it is important to better understand their scope and extent [8].

In the 1990s, the Faculty of Medicine and Health Sciences (FMHS) at Stellenbosch University initiated Community-based Education (CBE) as a strategy to train students appropriately for delivering primary health care services to South African communities [9]. In recognition of the need for more socially relevant training [10], undergraduate health professions students were sent to district and community health facilities (within the public healthcare system) as part of their clinical exposure. As this expanded, CBE came to be seen to include health service-based learning undertaken outside of the central academic hospitals, thus at distributed sites. A range of different experiences are now provided for all students across the five undergraduate programmes currently offered by the FMHS (dietetics, medicine, occupational therapy, physiotherapy, and speech, language and hearing therapy). At various times, students are placed in numerous public health facilities, under the supervision of a clinician who is already working at that facility. These sites include regional and district hospitals, community health centres and clinics, rehabilitation centres and hospices, and other non-governmental organisations, across three South African provinces (Western, Northern and Eastern Cape), although sites closer to the Faculty’s Cape Town base predominate.

Literature on the evaluation of clinical training at distributed sites is growing. Studies generally report positive experiences for students and communities [7, 11–13]. The approach has been shown to be associated with improved service delivery and quality of patient care, increased access to services for patients [14, 15], a positive change in hospital culture [16], patient and staff satisfaction and interprofessional collaboration [17]. It is suggested that this leads to improved health outcomes, along with greater health awareness and education, reported by communities [13, 18]. Academic involvement is also a source of motivation for local health services [19], with students seen to bring a sense of renewal and purpose to local practices [20]. Students training at distributed sites reported that they spend more time in clinical settings and have greater patient contact than is their experience in the central academic hospital settings [21, 22]. However, challenges have also been reported including inconsistent quality of training, inadequate facilities and supervision, and high financial costs [9, 11, 12]. This literature focuses on student, community and faculty experiences and perceptions about student training. There is less on the students’ contribution to the health facility and patient care. Evaluating outcomes and impact of programmes, and questioning whether patients are cared for differently as a result of students being placed at distributed sites [23] can be used to establish this.

This study provides insights from a number of health service-based training sites that differ in terms of geography, duration of placement, and professional programme, as well as from a range of different role-players (facility management; other staff at the facility; student ‘supervisors’ at the facility). We aimed to understand how the contributions of students are perceived by these key role-players, in order to foreground affordances, and in so doing promote greater interdependence between service and education to strengthen the provision of health service to the community.

## Methods

This study adopted an interpretivist approach, analysing qualitative data obtained during individual interviews held at eight public health care facilities in three provinces in South Africa where our students are placed. The project expanded on previous work that had been conducted amongst medical students as part of a multi-country study, using a similar data collection instrument [7] and sampling approach [7] to provide a fine-grained analysis across a broad range of sites and disciplines.

In 2015, all 80 distributed sites that were used for short clinical rotations (between 4 and 8 weeks), were approached regarding participation in the research project, through the respective provincial authorities. Permission was given by 53 sites. For the purposes of sampling, these were then stratified using a sampling grid designed according to a set of criteria applied proportionally to the current spread: provinces and districts; geographical location (urban, peri-urban, rural, deep rural)^1^; level of care (district or regional) and the mix of health professions students training at the site. Where more than one facility met a specific set of criteria in the grid, convenience sampling was followed to select one of these for the study. Eight sites, all of which had been receiving students for a number of years, were included in the final sample for analysis.

At each site, the health facility manager (M) responsible for day-to-day operations of the facility, one clinical supervisor (S) who provided direct clinical oversight of the students, and one other clinician (C) at the site who did not directly supervise the students, were invited to participate in the study. In the end, 24 interviews were conducted; at Site 1 the interview with the manager was repeatedly postponed due to work pressure and eventually withdrawn; at Sites 3 and 4, two supervisors were interviewed, while at Sites 1 and 6, all clinicians felt that they were involved in student supervision and we were, therefore, not able to identify an interviewee from the non-supervisor category (Table 1).

**Table 1:** List of included sites indicating location, nature of the facility, undergraduate programmes conducting training at the site, and the professions of the different interviewees

	Province	Level of care	Undergraduate Training Programmes(s)	No of interviews	Roles of interviewees	Area
1	Western Cape	District hospital	Medicine, Dietetics	2	Medical doctor (S); Medical doctor (C)	Rural (R)
2	Northern Cape	District hospital	Medicine	3	Manager (M); Medical doctor (S); Medical doctor (C)	Deep rural (D)
3	Western Cape	Community health centre	Medicine, Occupational Therapy	4	Manager (M); Medical doctor (S); Occupational therapist (S); Professional nurse (C)	Urban (U)
4	Western Cape	District hospital	Medicine, Physiotherapy	4	Manager (M); Medical doctor (S); Physiotherapist (S); Medical doctor (C)	Urban (U)
5	Eastern Cape	District hospital	Medicine	3	Manager (M); Medical doctor (S); Community service doctor (C)	Deep rural (D)
6	Western Cape	Regional hospital	Medicine	2	Manager (M); Medical doctor (S)	Peri-urban (PU)
7	Western Cape	District hospital	Medicine, Physiotherapy	3	Manager (M); Medical doctor (S); Physiotherapist (S)	Rural (R)
8	Western Cape	District hospital	Medicine	3	Manager (M); Medical doctor (S); Professional nurse (C)	Rural (R)

*M* manager, *S* clinical supervisor, *C* clinician

All participants gave written informed consent. Individual, face to face or telephonic, semi-structured interviews were conducted by a fieldworker although the interviews at Site 2 were conducted by one of the research team members not involved in the teaching of students. The interviews sought to generate in-depth information on their perspectives of and attitudes towards the presence of health professions students at their facility. Face to face interviews were conducted at the participant’s place of work. Telephonic interviews were conducted at Sites 5 and 8, as well as with one of the interviewees at Site 1 (Clinician = C). Interviews were conducted in English, audio-recorded, anonymized and transcribed. There was no discernible difference between the transcripts generated from the face to face interviews versus the telephonic interviews. The transcripts were subjected to thematic analysis [24]. All members of the research team participated in this iterative process to provide a rich and detailed, yet complex, account of the data [25]. Initial analyses per site and code lists were discussed and compared, after which the data were coded in a systematic fashion across the entire data set.

To enhance the trustworthiness and credibility of the study, data were triangulated by including participants who represented different activities on the training platform (i.e. Facility managers, clinical supervisors and clinicians) and by sampling different types of facilities from a range of geographical areas. Interviews with more than one individual at the same facility enhanced the rigor and transferability of results [26]. Ethical approval for this study was granted by the Health Research Ethics Committee at Stellenbosch University (reference N16/01/004).

## Results

Our focus was to examine the contribution made by undergraduate students to the distributed health care facilities in which they undergo a period of clinical training. In this section, the findings are presented in terms of the influence on the facility, including the staff at that facility, on patient care, on the communities in which the facilities are located, and on the clinical supervisors responsible for the students at the sites.

### Influence on the facility

Exploring issues relating to the influence that health professions students had on the health care facility foregrounded a number of factors both cultural and material. Having students at the site manifested in the organisational culture and the ways in which staff at the facilities engaged with one another. For example, respondents spoke about the way in which bringing the academic endeavour to the facility had a ripple effect on the culture in the facility by encouraging the adoption of a more evidence-based approach. This seemed to be felt across the facilities as the responses were not limited to those who worked directly with the students. One non-supervising respondent, for example, spoke of how it created a “nice academic environment” (4UC^2^) because there was a focus on teaching that had not been there before. A manager described how students asked “questions about things that you haven’t necessarily been thinking of for a few years” (5DM).

The fostering of an academic environment was not the only influence felt on clinical practice within the facility. For example, having students at the site who were on a Family Medicine rotation heightened awareness of adopting a more holistic approach to care and taking time to understand the patient’s context:

… they [the students] are thinking more of the sort of social contextual aspects. … and talking about the family and the context and all that kind of thing, it’s something that when you are just seeing numbers and things in … the outpatient department, you’re not necessarily taking the time to get all that contextual information (5DM)

The presence of students from different professions was also seen to change practice, particularly through encouraging inter- or multi-professional engagement.

… they need multidisciplinary input. So the rest of the multidisciplinary team actually then sits in on their presentations. So we will then make sure that the physio, the social worker, the rest of the team that is here are also present (8RS)

… just experiencing the awesomeness of working in a really well functioning team of professionals with everything from the allied health services to pharmacy. Everybody chips in, we are all part of the same community, we all get along (5DM)

There was a sense that the students were everyone’s responsibility, not only in terms of their learning, but also their wellbeing.

... we do feel that we take responsibility for their well-being while they are here…., and we need to assist them with that. So that’s what we perceive. (8RM)

With regard to more material issues, the use of consumables and resources, the overall sense from the supervisor respondents was that the impact was minimal and represented a valuable investment, noting that “it’s like having another doctor there. So whatever resources that person needs who is seeing a patient, those resources are needed anyway” (1RS).

I'm not going to use more physical resources. Perhaps if they’re not very good with putting up drips they might use one or two needles or syringes or whatever more, but I don't think it’s a train smash in terms of consumables (6PS)

So if you do a special investigation, like drawing bloods or whatever, or stitching or whatever you are teaching them, and they get it wrong through lack of experience, you might just need to use another blood tube or a Jelco® if they don't get a drip up. But those are like small, really small things, in terms of money. It’s not really big things. (8RS)

The presence of students was also seen to help relieve staff shortages, across the professions, as they can assist with important, albeit routine, patient-care related tasks, which are sometimes neglected due to staff and time constraints.

…they bring a lot of assistance to the professional nurses who are working in casualty… they assist a lot, especially with regards to clerking the patients, getting the history of the patients, which is something which on most occasions is very difficult to do, because of the shortage, because now we have to run against time… So they really bring a lot of relief for both the professional nurses and the doctors. (2DM)

The relationship between the university and the facility was foregrounded. There was a sense that while facility staff enjoyed having the students at the site, there were mixed reactions to the university that had sent the students. While some described a very positive experience, other comments related to poor communication from the university, particularly with regard to the specific learning outcomes set for the students.

… it’s just the efficiency and how well organised they are. We have never had a problem. It’s been an absolute pleasure to work with them [the university] … (7RS).

… the communication and the involvement in that has not been very good from the university’s side … especially in terms of the community project … (5DS)

### Influence on patient care and community

The respondents reported a range of activities that the students were involved in. In the health facility, for example, they assisted with history taking and examination of patients, the clerking of patients, dealing with emergencies, and performing certain simple procedures. The allied health students provided therapeutic interventions. In the community context, students conducted Quality Improvement and Community Oriented Primary Care projects, and were involved in health promotion, patient follow-up and home visits.

In performing these clinical activities, the respondents indicated that the students added to service delivery by doing more in-depth as well as comprehensive assessments of patients, being able to see patients more regularly than qualified clinicians, and assisting facility staff in patient care.

They take more time with patients, and they actually help you get a better history and understanding of patients, and also they do it according to a sort of family medicine way of interviewing a patient, which you sometimes forget to do that with all your patients when you are quite busy. So it was nice to just have them actually sit with patients and doing a more extensive history taking. (1RC)

… in a busy unit students tend to see patients more comprehensively, and they would then sometimes do a full assessment and spend time, especially in a busy EC unit, on specific patients, and then through the engagement with the patient elicit issues that wouldn't normally be picked up. (8RS)

The respondents said that the students had a positive effect on the patient workload at the health facility by using terms like: “more hands to do more detailed work” (3US2); “make work a bit lighter” (1RC); “helps us to see a little bit more clients” (3UC); and “help us doing practical stuff” (6PS); “I think positive, definitely because they see like between 13 and 20 patients, they see in a day extra that I couldn’t see by myself.” (7RS).

The effect of this more efficient and thorough service was seen as having a positive impact on the quality of care provided by the facility:

I would say that the quality of the treatments, the quality of our service does generally, it can be increased because there are more hands to do more detailed work and more detailed case studies, more detailed treatments. So I think generally it does promote the quality of our care, and not necessarily the numbers [of patients seen]. (3US)

As a result of the students’ activities in the community, the respondents indicated that the community recognised and appreciated the training institution’s presence in their community, and that the students served as aspirational role models especially for the youth in the community.

Unwittingly the student portrays something, and also the university is involved with the community. I mean, the University of Stellenbosch - that immediately creates an atmosphere. (1RS)

Respondents felt that patients welcomed the students and experienced the students positively. They described the patients as feeling valued and affirmed because students spent more time with them, taking time to explain, paying more attention, and attending to details. For example “patients actually feel like they’ve been listened to” (7RS); “the patients enjoy the students” (5 DC); and “they give that extra care to the patients… students have more patience with the patients also” (8RC).

Respondents thought that benefits for patients as a result of the students’ presence in the health facility included keeping patients informed, through improved patient education about their health conditions.

So it keeps our patients informed, and I think they also just gain more knowledge about the condition that they have. (3UC)

The issue of time was mentioned with some respondents saying that patient waiting times were less when there are students:

The waiting times would certainly be, I mean, if a student is there, the students see a patient, the EC waiting times will come down. The patient, instead of waiting 45 minutes, he will only wait 15 minutes and then be seen. (1RC)

On the other hand, some respondents thought that the presence of the students could increase the waiting time for the patients, as expressed in this quote from the manager of a facility:

I don't think it really affects the quality of care, except for maybe the waiting periods might increase because they spend more time with students around. But I think the quality of service is once they have seen the patient, that is definitely not compromised. It’s just that people have to wait longer. (6PM)

Nevertheless, a possible negative effect of students on patients was mentioned in that students might struggle with procedures:

Maybe the patient needs fluids and now you’ve tried five times and you still haven’t got any fluids or the antibiotics. (4US1)

In the community, the home visits and patient follow-ups were seen as particularly beneficial as they reduced the need for patients to go to the hospital, and improved patient outcomes. This enhanced the community’s perception of care “they (the community) just love seeing the [student] doctors”. (6PS)

Patients have benefitted because we try and get the students to do home visits, the patient that they see that become their patients, they are much more holistically treated. (5DS)

At one institution, the regular placement of students at the facility is viewed in such a positive light that their arrival is actually “advertised” in the local media!

Usually when the students, we don't do it with all of them, but when they arrive, we usually inform our local newspaper that we have got new students, and then a picture will be taken of them and put in the paper, so that when they visit the hospital, or even the clinics, they already know that these are the new medical students for this month. (2DM)

### Influence on supervisors

The common thread from supervisors was how much they enjoyed the opportunity to interact with students. This was often expressed in terms of conveying their own enthusiasm and interests to future health care professionals.

I just love them. I totally adore having students, because I’m passionate about medicine, and I can instil that into the students (6PS)

For the facility, I think for me, I see it as an opportunity to actually play a role in forming the type of medical professional that we would need to be able to cope with the demands of the services (8RS)

Supervisors and managers recognized this as bringing a difference to their work. While one suggested that difference was neither intrinsically good nor bad, others described enhanced job satisfaction and how an awareness of being “role models” (3US2) for the students prompted their own learning.

It increases staff job satisfaction, seeing as they have the opportunity to be part of teaching. So yes, we are lucky that we have them, we enjoy teaching. (8RM)

I just think when students are there we are more alert and more vigilant because we know that we’ve been watched …. usually by them asking you questions you will quickly realise that there is something that you’re not sure about and you will brush up on that. (7RS)

Another supervisor referred to this as a more widespread creation of “a learning environment” (3US2), with students keeping them “on their toes” (4US2) stimulating them to keep up to date with evidence, and positively influencing the supervisors’ perceptions of the standard of their own practice.

They’re stimulating and that conversation you need to be up to date, especially evidence based medicine, and that’s where they lift, indirectly, the standard of care. (4UM)

Having students also re-ignited forgotten passions:

[I am] taken back to a place where we feel motivated again…and it’s just made me realise again about rural medicine and why I did it and why I am here. (7RS)

An area of contention was time for teaching, which seemed to be perceived differently by managers and supervisors. Managers would refer to teaching as taking the clinician’s time away from “get[ting] through their day of clinical work” (6 PM), but on the other hand they could also see that “students definitely add to our efficiency” (4UM)

… it depends on what you mean with staff satisfaction … If it is on enjoying their [the clinical supervisors] work, it’s not a problem, [teaching is] part of it and they would enjoy it. If it is of getting through the day and all the work that you had to do …, then you feel good but if you are hampered by teaching along the way all the time and you don't get through the day then you can get quite stressed and then it could feel like it’s a burden. (7RM)

Supervisors’ perceptions seemed to depend on whether they saw teaching as something that is done when they are not busy with patient care, or whether they saw teaching as involving the student in their daily work activities.

My time is very limited because as I said I don't get designated time to do teaching…I don't get time to do tutorials and things like that. (7RS)

The thing is obviously to balance the supervision, tutoring, mentorship with the day to day work. So the day to day work doesn’t stand still, and many times, on the periphery, you are not allowed time off to spend with the students. It’s pretty much part of your day to day work. You’ve got to continue with that work and you’ve got to supervise. That is a challenge and the best way for me to overcome that was to involve students in the day to day work. (1RS).

In the latter case, students were seen as making a contribution to the supervisor’s clinical duties – assisting with procedures, helping with ward work, getting results. There was an acknowledgement that although students may seem to take from the supervisor’s time, they also helped in dealing with the work that needed to be done (the “brunt” (5DS)).

The supervisors’ opinions were influenced by the primacy of patient care and the need to ensure that care was not adversely affected by the patients possibly having to wait longer (see benefits for patients and community above).

## Discussion

This study provides further evidence for the potential benefit inherent in establishing distributed clinical training sites in addition to central academic hospitals. Generally the responses framed the experience as positive. As was the case with other recent work emanating from sub-Saharan Africa, this benefit was described as being felt by both staff and patients at the facilities, and having a value-add for the facility itself [3, 7]. This has particular relevance in the resource-constrained environment that characterises much of the health care system in the region. In this context, the potential to enhance the work environment particularly in fostering interpersonal and collaborative approaches to care, to lighten the workload, to encourage the adoption of evidence-based practices as the academic endeavor and service delivery coalesce, and to improve the quality of health care as well as the patient experience, cannot be underestimated. Also of interest was the fact that there was considerable alignment in responses across the range of facilities, despite the different professions represented. This suggests that while the contexts, and therefore the experiences, may differ, benefits are still seen to accrue for facilities, patients, communities and staff.

We were, however, keen to delve beyond what Elder-Vass [27] describes as a ‘level abstracted view’ to obtain a richer understanding of the mechanisms at play beneath what is perceived empirically. This, argues Elder-Vass [27], is the role of science and enables the researcher to expose the different layers of events and entities, and the interplay between them, that make up the event under scrutiny. It was evident from our data that there were clear consequences that emerged as a result of taking the clinical training of future health care professionals to distributed sites. We looked at these consequences in the context of five broad categories that were most dominant in our data, namely workload, patient experience, quality of care, teaching, and learning communities. We realised, however, that these consequences are reliant on certain conditions being present. In addition, further critical reflection pointed to a set of caveats or provisos that modulated the nature of the conditions. Table 2 provides an overview, with specific examples, of this stratified analysis across the five categories and emphasises the complexity inherent in this context.

**Table 2:** A stratified analysis of emergent events

Category	Consequence (manifests as …)	Conditions (if …)	Caveat (but …)
WORKLOAD	decreased workload	students are involved in everyday work activities	then students need to be more senior
PATIENT SATISFACTION	increased patient satisfaction as a result of, for example, shortened time to be seen in the emergency unit	students are “more hands”	lengthens time of each consultation because students take longer
PATIENT CARE	enhanced patient care	students are more thorough and holistic	this may be dependent on their skills and the nature of the supervision
TEACHING	job satisfaction and personal growth	teaching is not seen as a “burden”	then teaching should occur by involving the student in everyday work
LEARNING COMMUNITY	encouragement to update and deepen the supervisors’ clinical practice	the students bring the curriculum (e.g. evidence based medicine, biopsychosocial approach) to the team	this requires university support

The data from this study, across a range of distributed clinical training sites and five health professions programmes, shows unequivocally that students can make a positive contribution to health care services, on a number of levels. However, our stratified analysis suggests that such a contribution is dependent on certain critical factors (conditions) being in place. These include students integrating learning into practice, sharing responsibility for patient care, and taking time to be thorough in caring for patients. In addition, supervisors need to ensure that learning is part of everyday practice for the clinical team, which also shares in their students’ learning. In order to meet such conditions, issues (caveats) that have to be addressed include ensuring that students are more senior and thus more able to participate clinically, are given space and time to work, have adequate supervision, and are integrated into the work of health care, while the site and its staff require ongoing faculty support. The interconnectedness between the different categories is also evident, such as between patient satisfaction and patient care. However, inherent in this interconnectedness are possible tensions as taking more time with a patient may enhance the level of care they receive, but may lengthen the time spent at the facility. Thus, there exists a complex network of factors that academic institutions need to be mindful of when seeking to establish and maintain relationships at distributed sites. Such awareness might also facilitate clearer communication between the academic institution and the facilities that are integral to enabling successful clinical teaching.

While this study builds on work that has been ongoing at our institution since 2011 [2, 9, 16, 22, 28, 29], a limitation is that it remains a single-institution study. In addition, although there were no obviously divergent responses across the data, we did not specifically compare responses across the different contexts (rural, deep rural, urban or peri-urban) and this is an area for future investigation.

## Conclusions

This research set out to obtain insights from multiple stakeholders across a range of contexts, to understand how they experience having students training at their health care facilities. Our intention was to identify aspects of the placements that have benefits for the facilities, patients and communities that become involved in the training of health care professions students, in the hope that this might encourage greater interdependence between the health and educational systems [1]. If we accept that the successful implementation of clinical training on a distributed platform is reliant on such interdependent relationships characterised by symbiosis between the different roleplayers [2, 30], then this stratified analysis provides a framework within which we can understand the mechanisms at play within these relationships. This has relevance not only in South Africa, but also elsewhere in the world where there is a growing, indeed established, practice of training students outside of the central academic hospital [4, 7, 31]. We would argue that the findings from this study can inform future interactions between academic training institutions and health facility managers as well as regional, provincial and national departments of health. Ultimately, it points to a matrix of factors that need to be taken into account when the responsibility for training health professions students becomes distributed.

## Footnotes

1 In South Africa there is no formal classification system for these categories. The following was applied:

- **Urban** – metropoles and large towns (over 100,000 people)
- **Peri-urban** – settlements and small towns in the vicinity of cities that serve as commuter zones for large numbers of people who mostly work in the cities; they are concentrated often a legacy of apartheid spatial planning
- **Rural** – areas that are more than 100 km from the nearest city, predominantly agricultural and related activities, including subsistence farming, where people live in settlements, villages, small towns or regional centres less than 100,000 people.
- **Deep rural** – remote villages and small communities that are distant from towns and cities.

2 Label indicates as follows: 4 = site # (see Table 1); U = urban, P = peri-urban, R = rural, D = deep rural; M = manager, S = clinical supervisor, C = clinician working at the site (non-supervisor).
